# Operationalizing Digital Health Equity in Artificial Intelligence–Enabled Patient Decision Aids for Older Adults: Mixed Methods Study

**DOI:** 10.2196/89011

**Published:** 2026-06-29

**Authors:** Cindy Yue Tian, Xiaochen Yang, Kailu Wang, Annie Wai-Ling Cheung, Jonathan Chun-Hei Ma, Canjie Lu, Jasmine Cheuk-Ying Yu, Crystal Ying Chan, Jiamin Chen, Kun Ouyang, Ivan Wai-Kiu Lin, Tim Hung-Cheong Pang, Shi Zhao, Yingwei Wang, Eliza Lai-Yi Wong

**Affiliations:** 1 JC School of Public Health and Primary Care Faculty of Medicine Chinese University of Hong Kong Hong Kong China (Hong Kong); 2 Centre for Health Systems and Policy Research, JC School of Public Health and Primary Care Faculty of Medicine Chinese University of Hong Kong Hong Kong China (Hong Kong); 3 Faculty of Medicine Chinese University of Hong Kong Hong Kong China (Hong Kong); 4 Department of Computer Science and Engineering Faculty of Engineering Chinese University of Hong Kong Hong Kong China (Hong Kong); 5 School of Computing National University of Singapore Singapore Singapore; 6 Society for Community Organization Hong Kong China (Hong Kong); 7 School of Public Health Tianjin Medical University Tianjin China; 8 Department of Medical Humanities Tzu Chi University Taiwan Taiwan; 9 Center for Palliative Care Hualien Tzu Chi Hospital Taiwan Taiwan

**Keywords:** artificial intelligence, co-design, digital health equity, older adult care, patient decision aids, umbrella review

## Abstract

**Background:**

Artificial intelligence–enabled patient decision aids (AI-PDAs) hold promise for supporting older adults with chronic diseases in accessing personalized health information, clarifying preferences, and engaging in shared decision-making. Achieving equity in their design requires attention to the complex health care and digital contexts in which these tools are used. While the Digital Health Equity Framework (DHEF) provides a conceptual foundation, practical strategies for its application remain limited.

**Objective:**

This study aimed to identify equity-related determinants and generate actionable design strategies for applying the DHEF to AI-PDAs for older adults.

**Methods:**

A mixed methods study was conducted. Semistructured interviews were conducted with older adults living with hypertension and/or diabetes, health care providers, and medical students to explore equity determinants relevant to AI-PDAs. In parallel, a review of reviews synthesized existing evidence on approaches to addressing these determinants. Interview findings and review findings were integrated through an iterative mapping process conducted by the research team and refined through multidisciplinary expert consultation involving medicine, public health, social services, and computer science.

**Results:**

A total of 33 stakeholders were interviewed, including 15 older adults, 8 health care providers, and 10 medical students. Thirteen reviews were included in the umbrella review. The integrated synthesis identified equity determinants spanning individual, interpersonal, community, and societal levels across both the health care and digital environments, together with cross-level concerns related to algorithmic fairness. These findings informed 5 recommendations for equitable AI-PDA development: (1) co-design with end users to address their needs, (2) embrace relationship-centered design, (3) leverage community resources to improve support, (4) promote accessible and equitable artificial intelligence (AI) governance in society, and (5) enhance equitable AI through algorithmic fairness. Together, these recommendations provide practical guidance for design, pilot testing, implementation, and evaluation.

**Conclusions:**

By integrating stakeholder perspectives with synthesized review evidence, this study extends the DHEF from a primarily conceptual framework toward a more practice-oriented approach for AI-PDAs for older adults with chronic disease. Health care settings serve as a mediating sociotechnical context where AI tools may either support or constrain equitable care participation. The findings underscore the need for interdisciplinary collaboration to align technological innovation with equity-oriented design. Future work should focus on co-designed prototypes, real-world testing, and measurable equity outcomes.

## Introduction

Chronic diseases, including hypertension and diabetes, are major contributors to global morbidity and mortality [1]. Effective management of these conditions requires coordinating lifestyle modifications, medication adherence, and ongoing monitoring, highlighting the need for approaches that integrate patients’ values into evidence-based care. Shared decision-making (SDM) is widely promoted to achieve this goal. It is a collaborative process in which patients and clinicians jointly discuss and evaluate health care options to determine the most appropriate course of action for each patient [2].

Patient decision aids are structured, evidence-based tools that facilitate SDM in chronic illness management [3]. They outline available treatment options, present information on benefits and risks, and assist patients in evaluating these options against their personal values and preferences. Artificial intelligence (AI) now offers a paradigm to enhance the functionality and impact of traditional patient decision aids. AI-enabled patient decision aids (AI-PDAs) embed advanced computational techniques into digital platforms to synthesize individual patient data with large-scale clinical evidence. Moving beyond static information delivery, these tools offer dynamic, personalized support by tailoring explanations, generating individualized risk-benefit profiles, and strengthening clinician-patient dialogue [4-6].

Older adults may particularly benefit from AI-PDAs, given their high burden of multiple chronic diseases and challenges in processing complex health information and applying it to their own context. However, without inclusive and thoughtful design, these benefits may fail to be realized. As noted in the inverse care law [7], younger individuals are more likely to adopt digital interventions, drawing greater attention in digital health development. Consequently, the needs of older adults have received limited consideration. Even when included, older adults are often treated as a homogeneous group, with insufficient recognition of differences in digital literacy, cognitive ability, and readiness to adopt new technologies [8]. Such oversights risk reducing adoption and widening existing digital inequities in this population [9].

To address these concerns, there is a need for equitable AI-PDAs for older adults, which are intentionally designed to ensure fair access, usability, and benefit across diverse aging populations. The Digital Health Equity Framework (DHEF) [10] offers a useful conceptual structure for this purpose, guiding the identification of digital determinants of health at the individual, interpersonal, community, and societal levels. Previous discussions of this framework have mainly focused on the digital environment (eg, whether people have access to technology) and on a market environment (eg, whether the market supports digital health) [11-13]. Much less attention has been paid to the health care environment itself, especially to the factors that influence the health problems these tools target [14]. Addressing equity determinants of AI-PDAs in the health care and digital environments ensures that these tools are not only technically accessible but also effectively support equitable patient participation in health care decisions. Furthermore, while the DHEF has advanced conceptual understanding, practical approaches for operationalizing identified determinants to inform the development of digital tools remain limited [15,16]. This gap makes it challenging for designers to translate equity principles into concrete development strategies for these tools.

This study reports the predesign phase of developing an AI-PDA to support SDM among older adults with hypertension and/or diabetes in Hong Kong. We aimed to systematically identify equity-related determinants and generate actionable design strategies that can inform the AI-PDAs for older adult care. Specifically, the study pursued three objectives: (1) to identify equity determinants related to AI-PDAs in health care and digital environments through stakeholder interviews with older adult patients, health care providers (HCPs), and medical students; (2) to synthesize evidence-based strategies for addressing these determinants through an umbrella review; and (3) to integrate insights from both the interviews and the literature review into actionable recommendations that operationalize the DHEF. Although these recommendations were developed for older adults with hypertension/diabetes in Hong Kong, they may also inform the design and implementation of equitable patient-facing AI tools in similar settings and populations.

## Methods

This study adopted a mixed methods design combining stakeholder interviews, an umbrella review, and expert consultations to develop contextually grounded, evidence-informed recommendations for operationalizing the DHEF for AI-PDA for older adults.

### Stakeholder Interviews

From July to August 2025, we conducted semistructured interviews with 3 key stakeholder groups using purposive sampling. First, older adults aged ≥65 years living with hypertension and/or diabetes were recruited through outpatient clinics, community health centers, previous project contacts, supplementary social media advertisements, and community activities serving residents of deprived housing estates in Hong Kong. Recruitment sought variation in socioeconomic and demographic characteristics relevant to digital health equity, including sex, educational attainment, and household income. Second, health care professionals involved in chronic disease management, including early-career (<6 years of clinical experience) and senior practitioners (≥6 years), were recruited through the research team’s professional network. Third, senior medical students were recruited through the university’s medical school, as they may offer emerging perspectives shaped by recent exposure to digital and AI-enabled tools in medical training. Recruitment and preliminary analysis proceeded concurrently. Saturation was assessed separately within each stakeholder group through iterative review of interview notes, coding, and team discussion. Interviews were concluded when later interviews generated few or no new codes and yielded largely repetitive accounts that did not substantially alter the interpretation of emerging themes.

To distinguish determinants in health care vs digital environments, the interview guide comprised 2 sections (Multimedia Appendix 1). The first examined participants’ understanding and experiences of SDM. As AI-PDAs are intended to facilitate SDM, we treated the factors that shape equitable participation in SDM as relevant determinants of equitable engagement with AI-PDAs in the health care environment. The second explored views on AI-PDAs, including expected benefits and concerns about using these tools in the digital environment. All interviews were conducted in person in Cantonese, audio-recorded, and transcribed using OneDrive transcription software. The transcripts were then manually reviewed for accuracy by 3 researchers (JCHM, CL, and AWLC). Audio recordings did not include participants’ names and other potentially identifying information. Electronic files were stored on password-protected systems accessible only to the research team.

Thematic analysis was conducted using a hybrid deductive-inductive approach. The key terms under each level from the DHEF served as the main coding structure, with equity determinants mapped onto its 4 levels (individual, interpersonal, community, and societal) within 2 analytic domains: the health care environment and the digital environment. Where data revealed determinants not anticipated by the DHEF’s original formulation, additional codes were generated inductively and subsequently mapped onto the most appropriate DHEF level. Transcript coding was conducted independently by 2 researchers (CYT and XY), with coding differences discussed and resolved through consensus. Interviewers and coders hold public health backgrounds and had no prior personal relationship with participants before recruitment. We acknowledge that this disciplinary background may have oriented interpretation toward structural and system-level determinants. To mitigate this, codes and emerging themes were discussed within the research team to consider alternative interpretations and ensure balanced attention to both individual and structural factors.

### Umbrella Review

This targeted umbrella review was conducted to complement the qualitative interviews by synthesizing evidence-based strategies for addressing the equity determinants identified through the study. The literature search was conducted to identify reviews published from January 2022 to August 2025 in PubMed, MEDLINE, and ACM Digital Library. The search focused on 2 domains: AI-PDAs for chronic conditions (hypertension/diabetes) and barriers/solutions to digital health equity. For screening purposes, AI-enabled tools were defined as digital health tools incorporating an AI-related component, such as machine learning, natural language processing, predictive modeling, or adaptive decision logic, rather than conventional static or nonadaptive digital tools. Digital health equity was operationalized as issues related to equitable access, use, usability, engagement, or benefit from digital health tools, including barriers, facilitators, disparities, or strategies to support underserved populations. Studies were eligible if digital health equity was addressed as either a primary focus or a clearly identifiable secondary focus. Search terms combined controlled vocabulary and free-text keywords related to chronic disease management/hypertension/diabetes, digital health equity/health equity, and review using database-specific Boolean operators and syntax. Search algorithms are detailed in Multimedia Appendix 2. The search was supplemented with a general internet search via Google Scholar, a focused search on key authors, and citation and reference tracking.

Studies were included if they met the following criteria: (1) review articles using a transparent methodology (eg, systematic review, scoping review, meta-analysis, realist review, integrative review, or narrative review); (2) discussed AI-enabled digital tools for chronic disease management; (3) discussed digital health equity in the context of chronic disease management, including hypertension or diabetes; (4) published in English; and (5) available in full text. All records were imported into Rayyan (Rayyan Systems Inc) for screening. Two researchers (CYT and XY) independently screened titles and abstracts and then assessed potentially eligible articles at the full-text stage. Disagreements were resolved through discussion and consensus. The included studies were then independently reviewed and summarized by 2 researchers (CYT and XY). We did not conduct a formal methodological quality appraisal of the included reviews, as the purpose of this targeted umbrella review was to support thematic synthesis and framework operationalization rather than to assess intervention effectiveness or the certainty of the evidence. Similar to the interview process, thematic analysis was conducted, guided by the DHEF, to identify suggested strategies at each framework level. Where strategies were found to operate simultaneously across multiple levels rather than primarily at a single level, these were identified as cross-level interactions.

### Data Analysis and Recommendation Generation

**Figure 1:**
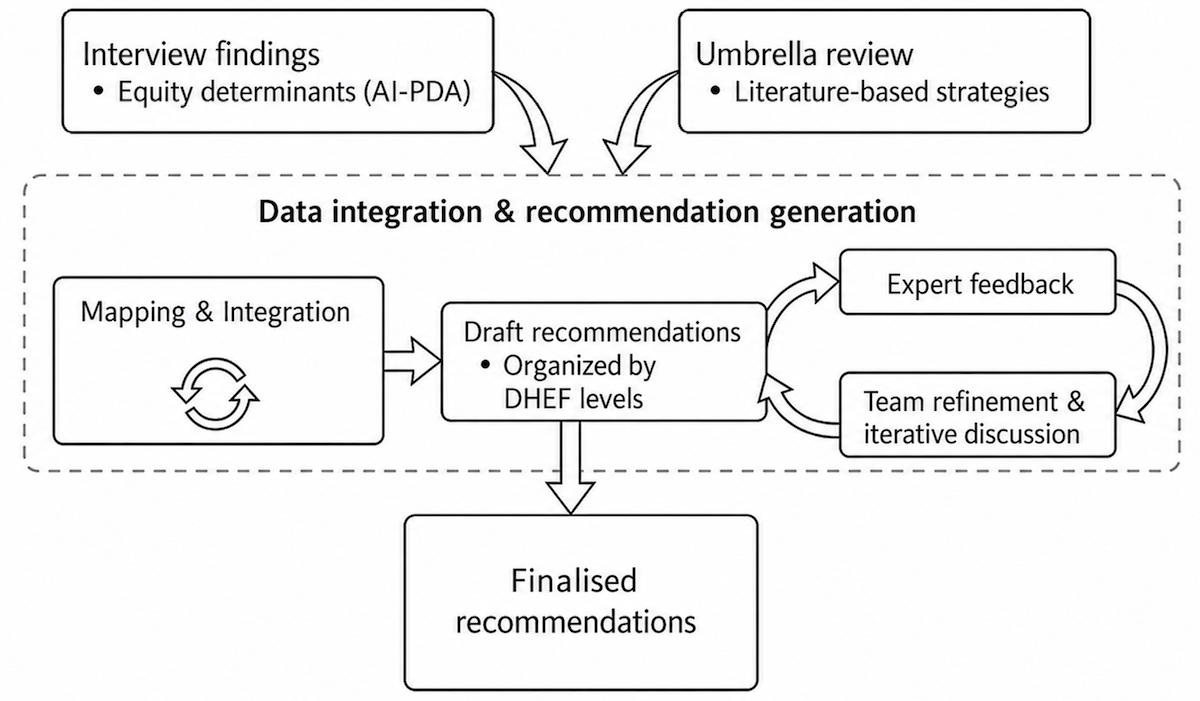
Study integration process for recommendation generation.

We identified determinants of AI-PDA equity from interviews and strategies from an umbrella review, both organized by DHEF levels (health care and digital environments). Similar to previous stakeholder-informed, evidence-integrated studies [17,18], the research team iteratively integrated the 2 sources (Figure 1). One researcher (CYT) first mapped the review-derived strategies onto the interview-derived determinants to identify alignment, additional considerations, and remaining gaps, and then prepared draft recommendations organized according to DHEF levels and cross-level considerations. These drafts were circulated to multidisciplinary experts by email for feedback and refinement. Feedback was incorporated through iterative discussion within the research team, with further consideration of expert comments where needed. After finalizing the recommendations, CYT added illustrative implementation priorities, primary stakeholder groups, and example indicators to support interpretation and practical application. These classifications were then shared with the multidisciplinary experts for final review and confirmation. The recommendations were therefore developed through iterative synthesis, team discussion, and expert input rather than through a formal consensus procedure.

### Ethical Considerations

Ethics approval for the stakeholder interviews of this study was granted by The Chinese University of Hong Kong Survey and Behavioural Research Ethics (reference number SBRE-24-0708). Written informed consent was obtained from all interview participants before data collection. Each participant received an HKD 100 (US $12.8) incentive in recognition of their time and contribution, consistent with local research practice and as approved by the ethics committee. The umbrella review and recommendation-generation involved evidence synthesis rather than direct human-subject research and therefore did not require separate ethics approval.

## Results

### Participant Demographics

Table 1 summarizes the sociodemographic characteristics of the participants, including 15 older adult patients with hypertension and/or diabetes (12/15, 80% with secondary education or below), 10 medical students, and 8 HCP. Except for 1 patient, all participants managed their conditions within the public health care system, so the identified determinants primarily reflect the public health care context. Table 2 presents the identified key equity determinants related to AI-PDAs from the interviews. Drawing on these qualitative insights and evidence from the 13 included reviews [13,19-30], we further engaged with a multidisciplinary advisory team (n=8; 2 experts each from medicine, public health, computer science, and social services) to propose actionable recommendations for operationalizing DHEF for older adults’ AI-PDAs. No major disagreements arose during the recommendation development process.

**Table 1:** Sociodemographic characteristics of the participants.

		Patients (n=15)	Medical students (n=10)	Health care professionals (n=8)
Age (years), mean (SD)		69.9 (4.4)	21.0 (0.9)	41.6 (12.0)
**Sex, n**				
	Male	9	5	6
	Female	6	5	2
**Educational attainment, n**				
	Secondary and below	12	0	0
	Postsecondary	3	10	7
**Household income, n**				
	Below median monthly household income	6	—^a^	—
	At the median monthly household income	5	—	—
	Over the median monthly household income	4	—	—
**Major, n**				
	Medicine	—	5	—
	Nursing	—	2	—
	Public health/gerontology/community health practice	—	3	—
**Expertise, n**				
	Specialist—family medicine	—	—	2
	Specialist—geriatric medicine	—	—	2
	Nurse	—	—	2
	Public health practitioners	—	—	2
**Clinical experience, n**				
	Less than 6 years	—	—	2
	6 years or more	—	—	6

^a^Not available.

**Table 2:** Key equity determinants of artificial intelligence–enabled patient decision aids (AI-PDAs) based on the Digital Health Equity Framework^a^.

Equity determinants of AI-PDAs	Health care environment	Digital environment
Individual	Limited health literacy; low self-efficacy; age-related cognitive decline	Limited digital literacy; low digital efficacy; distrust of AI^b^
Interpersonal	Paternalistic communication; weak patient-clinician relationship	Limited clinician engagement; provider technology bias
Community	Limited community support for SDM^c^	Limited community support for digital tools
Societal	Health system constraints; paternalistic medical culture	Affordability; regulatory gaps; poor health care integration

^a^This table is based on findings from the stakeholder interviews.

^b^AI: artificial intelligence.

^c^SDM: shared decision-making.

### Equity Determinants Related to AI-PDAs in the Health Care Environment

Key equity determinants affecting older adults’ use of AI-PDAs in health care are summarized below.

#### Individual Level

At this level, patients frequently reported limited health literacy (eg, “I don’t know how to ask my doctor question” [P2]), low self-efficacy (eg, “I don’t have much to say to the doctor because your level is different from the doctor’s” [P7]), and age-related cognitive decline (eg, “I forgot to ask the doctor... I had a lot of medications last time” [P9]), as determinants related to participation in SDM. Health care professionals and medical students also recognized these issues. They frequently described a subset of older adults who were more adept at seeking health information, asking relevant questions, and actively participating in consultations as smart. They noted that this group was more likely to be involved in SDM.

#### Interpersonal Level

Older adult patients often attend medical consultations without family accompaniment, making the patient-clinician relationship central in SDM. Two key determinants emerged at this level. First, clinicians’ paternalistic communication. Patients reported that doctors rarely presented multiple options; instead, communication often took the form of informing patients of a decision that had already been made (eg, “The doctor just informed you of the treatment, no space for me to make a choice” [P8]). Second, a weak patient-clinician relationship. Some patients expressed reluctance to voice preferences or concerns due to fear of negative attitudes from doctors. For example, one patient (P10) explained, “If I ask too many questions, the doctors will get annoyed.” By contrast, a patient treated by a physician recommended by a trusted church member described a more positive experience. She shared: “Because I knew the doctor quite well, I usually asked more questions” (P3).

#### Community Level

Patients faced challenges at the community level, where infrastructure to support SDM was largely absent (eg, patient education workshops related to SDM). With limited guidance on how to participate in decision-making, all patients often turned to YouTube, newspapers, and TV to address their health concerns, while community centers and official government resources were seldom consulted. This lack of SDM training was also observed by the medical community. Medical students reported limited exposure to SDM concepts, noting that curricula emphasized biomedical knowledge over patient engagement: “In class, the topic was not explained in depth, just briefly mentioned” (MD1).

#### Societal Level

Systemic issues related to the health care system and social norms emerged as important determinants at this level. Limited consultation time and fragmented continuity of care constrained both patients and providers. For example, one patient described the time pressure during consultations: “You wait for an hour, and then just get five or six minutes for the consultation” (P11). Health care professionals (HCP2, 3, 4) similarly reported frustration at being unable to provide comprehensive care under these constraints. Social norms, particularly a historically paternalistic medical culture, also influenced experiences. The majority of patients reported that they respected authority and simply followed doctors’ instructions.

### Equity Determinants Related to AI-PDAs in the Digital Environment

Key equity determinants influencing older adult patients’ adoption of AI-PDAs in the digital environment are summarized below.

#### Individual Level

Patients often reported that their use of AI-PDAs was strongly shaped by limited digital literacy (eg, “I didn’t know a mobile app could record blood pressure” [P4]), low digital self-efficacy (eg, “I’m just not good with technology” [P9]), and age-related cognitive decline (eg, “I always forgot my log in password” [P10]). A few patients (P3, P5, P10) tended to express their distrust in AI, particularly regarding the reliability of data sources and the readiness of new technologies for clinical use.

#### Interpersonal Level

Limited clinician engagement may hinder older adults’ use of AI-PDAs, as clinicians may be skeptical of AI and concerned that AI-generated information. As one nurse explained, “While the AI might provide information about ten different treatment medication options, then the patients have 10 questions, if so... we won’t use this tool because we don’t have time to address all these questions” (HCP4)*.* One patient (P10) expressed concern about no feedback from the clinicians if he used the tool. In addition, implicit technology bias can shape both provider attitudes and patient experiences. Some health care professionals assume that older adults are inherently unable to use digital tools, as reflected in one comment: “Even if you teach them how to use an app once, they might forget by the second time“ (HCP6). However, other professionals recognize this bias and emphasize that older adults can engage digitally when provided with adequate support. “There’s this bias that they can’t use digital tools, but in reality, they can—it’s just that they need support and facilitation. With that, they might actually manage it” (HCP5). Finally, the presence or lack of technological assistance from family members or caregivers also plays an important role. One patient described how her daughter, a nurse, taught her how to use the government’s digital tool to make a medical appointment, which enabled her to navigate the system effectively (P2).

#### Community Level

Limited community support for using digital tools is a crucial determinant. Among the digital health tools most familiar to older adults is the government’s official apps. Patients reported that they usually downloaded and learned to use the app during community-based promotion activities or workshops, which provided them with initial guidance and confidence in adoption. A public health care practitioner (HCP6) noted that assisting older adults with a digital platform for booking medical consultations was particularly beneficial for this population.

#### Societal Level

Many patients reported that affordability (eg, “I wouldn’t use the app if it’s not free” [P7]) would affect their use of this tool. HCPs emphasized that poor incorporation of AI-PDAs into health care systems would lead to underuse and limit equitable adoption. As one clinician (HCP1) shared, he collaborated with a research team to use an app for tracking patients’ hypertension. Typically, he would ask patients to show their blood pressure records through the app during consultation, which helped patients gradually become familiar with using it. He commented, “You really need to engage doctors and embed the tool into clinical workflows; otherwise, patients won’t use it.” Regulatory gaps represent another crucial determinant. Inadequate regulatory oversight may lead to variability in the quality of health information provided by these tools, undermining stakeholders’ trust and fair uptake. As one professional cautioned, “Be careful with AI, as its outputs may not always be consistent. You might get different answers today compared to tomorrow.” (HCP1).

### Strategies for Advancing Digital Health Equity From an Umbrella Review

**Figure 2:**
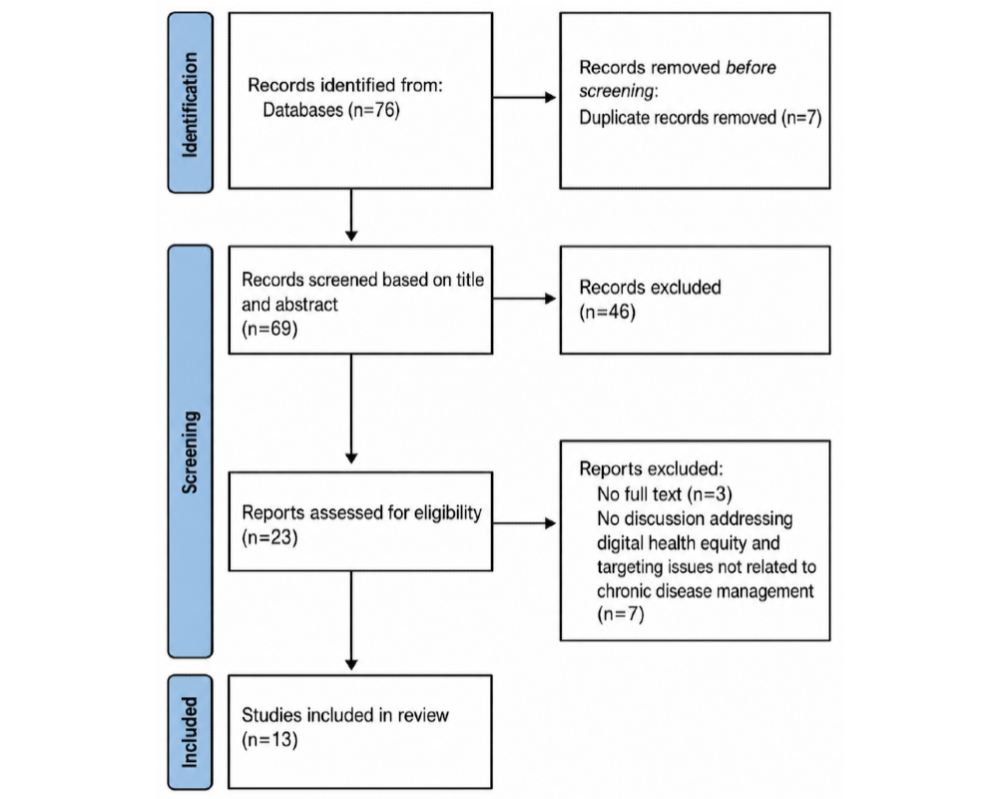
PRISMA (Preferred Reporting Items for Systematic Reviews and Meta-Analyses) flow diagram.

In the umbrella review, 13 identified reviews [13,19-30] were included (Figure 2). Strategies were synthesized across DHEF levels (Multimedia Appendix 3). Although the DHEF does not explicitly include a technology level, technology-related factors intersect with individual, interpersonal, community, and societal determinants, supporting a cross-level perspective in examining digital health equity. Technology-related determinants were most frequently represented in the review literature (n=26). In contrast, determinants at the individual (n=6) and interpersonal levels (n=4) were comparatively underrepresented, while community-level (n=10) and societal level (n=12) determinants received moderate attention. These frequencies indicate the degree of emphasis placed on each DHEF level in the existing literature rather than the relative importance of the corresponding strategies.

### Recommendations on Operationalizing the Digital Health Equity Framework

#### Overview

By mapping interview-derived determinants onto a synthesized evidence base from reviews, and engaging a multistakeholder team, we generated a set of actionable recommendations for applying the DHEF to the design of AI-PDAs for older adults (Table 3).

**Table 3:** Key recommendations on operationalizing Digital Health Equity Framework (DHEF) in artificial intelligence–enabled patient decision aids (AI-PDAs) for older adults^a^.

DHEF level	Recommendation	Key actions	Implementation priority	Primary stakeholders	Example indicators
Individual	Co-design with end users to address their needs	Tailor content to varying health and digital literacy levels; incorporate features that support self-efficacy and digital confidence; accommodate age-related cognitive needs through accessible design and communication.	Early-stage (design and pilot testing)	Developers, clinicians, patients	Subgroup uptake, onboarding completion, comprehension, need for assistance, self-efficacy, and sustained use
Interpersonal	Embrace relationship-centered design	Involve clinicians in design and workflow integration; maintain human oversight of AI^b^-generated recommendations; support participation of caregivers and family members where appropriate.	Early-stage (design and pilot testing)	Developers, clinicians, service managers, patients, caregivers	Clinician and caregiver acceptability, quality of communication during consultations, and perceived workflow burden
Community	Leverage community resources to improve support	Engage community partners in co-design and evaluation; provide community-based digital health support programs; connect users with trusted local resources.	Medium- to long-term (implementation and scale-up)	Community organizations, clinicians, service managers, policymakers	Participation in community-based support activities, sustained use with community assistance, perceived cultural relevance, and feedback from community stakeholders
Societal	Promote accessible and equitable AI governance	Reduce financial and technical barriers; establish standards for safety, transparency, and accountability; integrate AI-PDAs into routine care pathways; foster multidisciplinary collaboration.	Medium- to long-term (implementation and scale-up)	Policymakers, regulators, health system leaders, service managers	Affordability, staff training completion, integration into routine workflows, reported safety or escalation events, and institutional readiness for sustained implementation
Cross-level	Enhance equitable AI through algorithmic fairness	Ensure representative and inclusive datasets; implement bias mitigation throughout model development and deployment; support transparent reporting and equity monitoring.	Foundational technical standard (from design onward and remain central during postdeployment evaluation)	Developers, data scientists, regulators, institutional leaders	Subgroup-specific uptake and outcomes, fairness performance reporting across key older adult subgroups, documentation of bias mitigation steps, transparency of model limitations, and stakeholder-informed review of equity implications

^a^Content is based on integrated insights from stakeholder interview findings and the umbrella review, synthesized by the research team.

^b^AI: artificial intelligence.

#### Recommendation 1: Co-Design With End Users to Address Their Needs

Primarily directed to developers, clinicians, and patients, this recommendation emphasizes co-design and participatory approaches to ensure that the digital health solution reflects older adults’ needs, capabilities, and preferences [21]. Key strategies include:

- Tailor content to patient health and digital literacy: health literacy and digital literacy are key determinants of effective AI-PDAs, yet they are often overlooked in personalized interventions for chronic disease management [19,20,31]. Designers should first understand end users’ health and digital literacy levels, then adapt content to match these varying levels [20]. For example, provide simplified explanations of medical terms, step-by-step guidance, and longer training sections for beginners [13], while offering advanced features and data visualization for more experienced users.
- Psychological support to build self-efficacy and digital self-efficacy: psychological support is a potential mediator in building strong patient-clinician relationships and fostering sustained engagement with digital health tools for older adults [28,30]. Empathetic AI-enabled tools can provide encouraging feedback, a sense of progress, and tailored emotional guidance, enhancing older adult patients’ confidence in using these tools and helping alleviate stress associated with chronic illness and digital technologies.
- Support cognitive needs: AI-PDAs should accommodate age-related cognitive challenges through simplified interfaces, larger fonts, reminder messages, and voice guidance [28]. Additionally, present information in plain language and embed it in practical, everyday contexts to enhance comprehension and usability [22,24,28].

This recommendation may be considered an early-stage implementation priority as these strategies are often the most immediately actionable during design and pilot testing, although they remain important during implementation, and illustrative indicators of success include subgroup uptake, onboarding completion, comprehension, need for assistance, self-efficacy, and sustained use.

#### Recommendation 2: Embrace Relationship-Centered Design

This recommendation requires close collaboration among designers, clinicians, service managers, patients, and caregivers, as AI-PDAs are inherently situated within a network of interactions among patients, HCPs, and caregivers. Their design must explicitly account for these relational dynamics. Key strategies include:

- Engage clinicians in AI tool design: involve HCPs in co-design to ensure AI-PDAs complement clinical practice [28,30]. This engagement also raises awareness of the diverse digital capabilities of older adults, helping address clinicians’ implicit technology bias and strengthening clinician-patient relationships.
- Reinforce human-in-the-loop integration: clinicians are embedded within the AI-PDA workflow, actively interacting with patients while reviewing and interpreting AI-generated recommendations [19]. This ensures that AI-PDAs support, rather than replace, critical clinical judgment, thereby enhancing clinicians’ and patients’ trust in these tools.
- Support social interdependence: acknowledge that digital tool usage is often a collaborative activity for older adults. AI-PDAs’ architectures should therefore facilitate supported use by integrating functionalities for caregivers and family members to participate in data entry, progress monitoring, and decision support [28,30].

This recommendation, such as Recommendation 1, may also be considered an early-stage implementation priority, and illustrative indicators of success include clinician and caregiver acceptability, quality of communication during consultations, and perceived workflow burden associated with integrating the AI-PDA into routine care.

#### Recommendation 3: Leverage Community Resources to Improve Support

Primarily relevant to clinicians, service managers, community organizations, and policymakers, this recommendation recognizes that community-level determinants shape the adoption and effective use of AI-PDAs. Key strategies include:

- Engage community partners in co-design: engage community representatives (eg, nongovernmental organizations, faith-based organizations, and lived experience advisors) in co-design and usability testing, thereby linking AI-PDAs to trusted community resources. Community members can also contribute to study recruitment and ensure that diverse voices are represented [22].
- Mobilize community-based adoption support: facilitate adoption of AI tools through community-based initiatives, including health literacy, digital literacy, and AI literacy programs for patients, caregivers, and health care workers (eg, workshops or “tech buddy” programs) [22,30].
- Include community perspectives in ongoing evaluation: engage community representatives in the ongoing evaluation of AI-PDAs and longitudinal design to ensure cultural relevance and equitable outcomes [21,25,32].

This recommendation is more likely to be considered as medium- to longer-term priorities as these strategies depend on external coordination with community partners and sustained engagement beyond the initial pilot phase, and illustrative indicators of success include participation in community-based support activities, sustained use with community assistance, perceived cultural relevance, and feedback from community stakeholders.

#### Recommendation 4: Promote Accessible and Equitable AI Governance in Society

This recommendation depends particularly on policymakers, regulators, health system leaders, and service managers, as equitable implementation requires social support and governance mechanisms to reduce access barriers and guarantee the ethical integrity of AI tools. Key strategies include:

- Ensure affordability and accessibility: ensure AI-PDAs are financially and physically accessible to all patients, including those from disadvantaged groups [13]. This may involve securing financial resources or policy-driven subsidies to provide low-cost or free access, minimize data or download requirements, and reduce complex log-in procedures with particular attention to the needs of older adults [32].
- Design standards to ensure credibility and equity: the design standards aim to guarantee responsible and technically reliable AI tools by mandating safety, reliability, transparency, and accountability to prevent disproportionate harms across diverse populations [23]. For example, these standards should require clear communication about information sources, provide references to high-quality medical resources [25], data protection [28], and emphasize equitable health care access [24].
- Integrate into health care system workflows: ensuring systemic connections and clinical sustainability is crucial [24]. Identify suitable adoption settings, such as primary, secondary, or tertiary care, and embed AI-PDAs into existing workflows. Examples may include use before the consultation during nurse-led preassessment, in waiting areas through tablets, and during chronic disease follow-up in community health care centers [28]. Additionally, implementation should be accompanied by training and guidance for health care professionals to ensure effective adoption while maintaining workflow efficiency and high-quality care [28].
- Multidisciplinary collaboration: AI designers must recognize how technology intersects with this complex historical and social context [21]. Effective digital implementation in AI tools needs a strong public and private partnership and multidisciplinary collaboration, where nongovernmental organizations, private companies, academic institutes (eg, engineering, informatics, and social sciences), and government can collaborate to leverage each other’s strengths [19,32]

This recommendation is also more likely to be considered a medium- to longer-term priority as it depends on broader institutional, regulatory, and system-level changes, and illustrative indicators of success include affordability, staff training completion, workflow integration, safety or escalation events, and institutional readiness for sustained implementation.

#### Recommendation 5: Enhance Equitable AI Through Algorithmic Fairness

Primarily directed to designers, engineers, data scientists, regulators, and institutional leaders, this recommendation addresses data practices, model development, and reporting and governance processes. While technical implementation of fairness methods may be led by developers and data scientists, clinicians, patients, caregivers, and community partners contribute primarily through oversight, contextual interpretation, and identification of equity concerns in real-world use. The strategies below align with existing AI governance and fairness guidance, while highlighting considerations that are particularly relevant to AI-PDAs for older adults with chronic disease:

- Representative and inclusive data practices (developers, data scientists, and institutions): datasets should represent diverse populations across age, sex, race/ethnicity, and socioeconomic status, particularly including historically disadvantaged subgroups [19,25]. As the field evolves to use novel data from wearables and sensors, proactive bias mitigation is essential to identify and counteract new forms of digital usage bias inherent in these sources [19]. Sampling limitations should be transparently documented [20], and data privacy and security should be safeguarded throughout model development and implementation [29].
  Distinctive consideration for older adults’ AI-PDAs: datasets should capture heterogeneity among older adults, including age subgroup, health and digital literacy, caregiver dependence, health status, and living setting [20,24]. Recruitment and data collection should avoid single-mode strategies (eg, digital-only and clinic-only) that may systematically exclude more vulnerable groups [26].
- Fairness-aware model development and evaluation (developers and data scientists): this begins with preprocessing techniques such as oversampling underrepresented groups or applying importance weighting to mitigate initial data biases [21]. In-process techniques are then critical, directly embedding fairness into the model’s training through methods like parity constraints, fairness regularization, and adversarial debiasing to prevent the algorithm from learning discriminatory patterns [21]. Finally, postprocessing adjustments, such as calibrating model thresholds or test-retest for different subgroups, ensure equitable outcomes [21,23].
  Distinctive consideration for older adults’ AI-PDAs: in chronic disease management for older adults, false negatives and false positives may carry different clinical and psychosocial consequences across subgroups. Fairness assessment should therefore extend beyond statistical parity to include subgroup-specific evaluation of clinical impact, stakeholder-informed equity review, and threshold calibration based on differential risks, benefits, and implementation burden [26,29].
- Transparent equity reporting and governance (developers, institutions, and regulators): this includes (1) adherence to established AI-specific reporting guidelines (eg, Consolidated Standards of Reporting Trials-Artificial Intelligence [33] and Transparent Reporting of a Multivariable Prediction Model for Individual Prognosis or Diagnosis-Artificial Intelligence [34]) in all publications [19]; (2) public disclosure of equity impact assessments, including subgroup-specific performance metrics [24,26]; (3) formally incorporating and documenting feedback from diverse stakeholders to identify and address potential biases; and (4) explicitly communicating the AI’s intended use, capabilities, and limitations to end users to support appropriate use and prevent misuse [20].
  Distinctive consideration for older adults’ AI-PDAs: reporting should also support understanding and trust through plain-language explanations, disclosure of developers and evidence sources, documentation of input from older adults and caregivers, and attention to cultural, linguistic, and dialect diversity [25].

Algorithmic fairness should be embedded throughout the AI life cycle, with ongoing evaluation of subgroup performance, transparency of model limitations, and stakeholder-informed review of equity implications.

## Discussion

### Principal Findings

Although broader equity-oriented checklists have been proposed for AI and digital health, few studies have translated the DHEF into practical recommendations for developing AI-PDAs for older adults. This study addresses this gap by providing DHEF-informed recommendations tailored to this context. Drawing on stakeholder interviews and an umbrella review, we generated recommendations across 5 socioecological levels to guide more equitable design and implementation. This framing reflects the growing recognition that advancing equity-oriented digital health design requires moving beyond purely technology-centered or user-centered approaches [14,35,36]

Across the recommendations, a key finding was that equity risks emerged at both relational and structural levels. Stakeholder interviews gave particular prominence to individual- and interpersonal-level determinants, including trust, communication, caregiver involvement, emotional support, and confidence in using digital tools. These issues received comparatively less attention in the identified reviews, consistent with previous evidence that equity-related factors at individual and interpersonal levels are often underexamined in digital health research [11]. This gap is particularly important for older adults, for whom encouragement, trust, and support from clinicians, caregivers, and social networks can shape whether digital tools are perceived as usable, relevant, and safe [8,37]. 

At the same time, the umbrella review more strongly captured upstream community-, societal-, and technical equity risks, including governance, affordability, participatory design, data representation, and algorithmic fairness. We interpreted this divergence between stakeholder interviews and literature review as reflecting the comparative strengths of each evidence source rather than a conflict to resolve: stakeholders captured equity barriers as experienced in lived care interactions, while the literature captured upstream structural risks. These map onto distinct levels within the DHEF. During the iterative mapping process, literature-derived upstream concerns were retained and formalized into Recommendations 3, 4, and 5, even where stakeholders did not raise them. Conversely, stakeholder-identified relational determinants, such as human-in-the-loop integration and caregiver role-based access, were prioritized in Recommendations 1 and 2, even where literature coverage was limited. This level-sensitive integration is important because equitable AI-PDAs for older adults require both relationally responsive design and broader institutional, community, and policy support [38-41].

### Implications

Building on this multilevel interpretation, a central contribution of this work is to reconceptualize digital health equity in patient-facing AI as a sociotechnical and relational problem, not simply a question of access, connectivity, or algorithmic performance. Prior digital health equity frameworks, including the DHEF and related multilevel syntheses [42,43], have been valuable in identifying key determinants across policy, system, community, interpersonal, and individual levels, particularly with respect to general foci on access, connectivity, digital literacy, and digital inclusion. However, they have provided less explicit guidance on the mechanisms through which these determinants shape the real-world use of patient-facing AI in clinical encounters already marked by unequal communication, limited consultation time, workflow constraints, and variable caregiver involvement. Our findings suggest that the health care environment is not merely a background context, but the mechanism through which digital tools are either converted into meaningful participation in care or fail to generate benefit. In this sense, AI-PDAs do not enter an equity-neutral setting; rather, they are introduced into encounters already structured by disparities in health literacy, clinician-patient communication, trust, caregiver dependence, and system-level access barriers affecting older adults. This shifts the equity question from whether patients can access AI to whether they can actually use it to gain understanding, exercise agency, and influence decisions within real consultations. The implication is that equitable AI implementation requires more than technically robust tools. It requires workflow-sensitive, relationship-aware, and institutionally grounded design that accounts for how benefits are mediated, amplified, or constrained in practice [44,45].

Importantly, we position AI algorithmic fairness as a cross-level domain, rather than a separate level, because the DHEF does not specify a stand-alone “technology” level [10]. Rather than functioning independently, algorithmic fairness is shaped by and operates across social and structural conditions throughout the framework. For AI-PDAs for older adults, fairness cannot be treated as a downstream concern to be checked after deployment. Instead, it requires early and ongoing attention to data representation, bias mitigation, subgroup-specific evaluation, and transparent reporting across the full AI life cycle. This is particularly important because older adults are heterogeneous, and inequities may arise not only across conventional demographic categories but also across differences in health literacy, digital literacy, caregiver dependence, health status, and living setting [46,47]. By integrating user-centered and technical equity considerations, this work provides practical, cross-disciplinary guidance for advancing equitable AI implementation in health care [48-50].

While the multilevel logic of the DHEF provides a transferable organizing structure, the specific recommendations were developed in the Hong Kong context and therefore require contextual adaptation before implementation elsewhere. Hong Kong’s predominantly public-led health system, marked by high service demand, time-constrained consultations, and particular care pathways, likely shaped both the need for and the potential role of AI-PDAs. Transferability may also be influenced by local cultural norms surrounding medical authority, family involvement in care, and attitudes toward technology. For example, in settings where health decisions are shaped more collectively through family involvement, the adaptation of AI-PDAs should consider who is expected to use the tool, whether caregivers should be included in the decision-support process, and how patient autonomy and caregiver input can be balanced. In contexts where patients are less accustomed to questioning clinicians or using self-directed support tools, implementers should consider whether AI-PDAs need to include additional guidance on preparing questions, clarifying preferences, and initiating SDM conversations. In addition, Hong Kong’s dense urban environment and relatively strong digital connectivity may facilitate access and implementation in ways that do not readily translate to settings with more dispersed populations, weaker digital infrastructure, or greater barriers to continuity of care. In such settings, implementers should consider whether older adults have reliable access to devices, connectivity, and support, and how AI-PDAs can be embedded into existing community or primary care workflows.

Accordingly, the key issue is not whether these recommendations are transferable in principle, but how their relative priority, sequencing, and feasibility should be adapted across settings. For example, in low- and middle-income countries, Recommendations 3 and 4 may need to place greater emphasis on foundational infrastructure and community health worker integration before institutional deployment, with explicit attention to the training and governance arrangements this requires [51,52]. Similarly, in settings with different cultural attitudes toward aging, medical authority, and family involvement, Recommendations 1 and 2 may need recalibration to reflect local relational norms [53]. For instance, where family members play a more central role in health decision-making, or where deference to clinical authority shapes how patients engage with self-management tools. Future research should therefore test and refine these recommendations across diverse health systems, sociocultural, and digital contexts.

### Limitations

Limitations of this study include the following. First, although we followed PRISMA (Preferred Reporting Items for Systematic Reviews and Meta-Analyses) guidelines for our literature review, some relevant studies may have been omitted due to subjective judgments during the selection process. Second, we only included studies published in English, potentially limiting the comprehensiveness of our analysis. Third, we did not formally assess the overlap of primary studies across the included reviews; accordingly, the reported frequencies reflect how often strategies appeared in the review literature rather than the number of unique supporting studies. Fourth, we did not conduct a formal methodological quality appraisal of the included reviews; therefore, the synthesized strategies should be interpreted as descriptive review-level findings rather than as evidence of equal strength across recommendations. Fifth, the classification of suggested strategies by level involved subjective judgment by the research team. While we applied systematic procedures and multidisciplinary discussions to enhance consistency, some degree of subjectivity may have influenced the categorization. Future work could involve in-depth consultations with AI tool developers to gain additional insights on equity determinants. Sixth, these recommendations, although informed by stakeholder perspectives and the umbrella review, have not yet been empirically tested as formal design requirements or evaluated for their effects on digital health equity outcomes. They should therefore be considered preliminary, evidence-informed design considerations. Seventh, the recommendations were refined through iterative email feedback from multidisciplinary experts rather than through formal consensus meetings or a structured consensus method. Although this pragmatic approach supported multidisciplinary input, it may have limited reproducibility and reduced transparency in how differing perspectives were weighed and how priorities were established. Finally, social desirability bias may have influenced the findings, as clinicians may have underreported paternalistic practices and patients may have minimized dissatisfaction or hesitated to express criticism. Although we sought to mitigate this through semistructured interviews and cross-stakeholder comparison, this bias cannot be fully excluded.

### Conclusion

This study operationalizes the DHEF by translating its concepts into practical strategies for designing inclusive AI-PDAs for older adult care. The findings suggest that health care settings function as a mediating sociotechnical context through which AI tools may either support or constrain equitable participation in care. Consequently, the study underscores the necessity of interdisciplinary collaboration encompassing technology designers, clinicians, public health professionals, social service providers, and policymakers to reconcile technological innovation with user-centered and equity-oriented design. Future work should focus on translating these recommendations into co-designed AI-PDA prototypes, testing them in real-world clinical settings, and establishing measurable equity-relevant outcomes and process indicators to evaluate whether implementation is both effective and equitable.

## Data Availability

The datasets generated and analyzed during the study are not publicly available due to the inclusion of potentially identifiable information from stakeholder interviews, but are available from the corresponding author on reasonable request, subject to ethical approval and data sharing agreements. The review of reviews included publicly available literature, as cited in the manuscript.

## Appendix

Multimedia Appendix 1 Overview of interview domains and illustrative questions.

Multimedia Appendix 2 Search algorithms of the umbrella review.

Multimedia Appendix 3 Summary of suggested strategies for addressing digital health equity from the included reviews.

## Abbreviations

AI: artificial intelligence
AI-PDA: artificial intelligence–enabled patient decision aid
DHEF: Digital Health Equity Framework
HCP: health care provider
PRISMA: Preferred Reporting Items for Systematic Reviews and Meta-Analyses
SDM: shared decision-making
