# Supplementary material for: Operationalizing Digital Health Equity in Artificial Intelligence–Enabled Patient Decision Aids for Older Adults: Mixed Methods Study
Source: J Med Internet Res. 2026 Jun 29;28:e89011. doi: 10.2196/89011 (PMC13365886; doi:10.2196/89011)
Supplement: Multimedia Appendix 1 [file jmir_v28i1e89011_app1.docx]

**Supplementary Table S1.** Overview of interview domains and illustrative questions

| **Environment** | **Domain** | **Illustrative question** | **Example probe** |
| --- | --- | --- | --- |
| Healthcare environment | Chronic disease management experiences | What concerns do older adults have regarding management of hypertension or diabetes? | Medication use, complications, self-management challenges |
|  | Treatment decision-making experiences | How do patients typically make treatment decisions? | Independent decisions, family involvement, reliance on clinicians |
|  | Shared decision-making experiences | How involved are patients typically in treatment decisions? | Active participation vs reliance on clinicians |
|  | Barriers to participation | What challenges affect patients’ ability or willingness to participate in treatment decisions? | Health literacy, digital literacy, time constraints, language and cultural factors |
|  | Information seeking and decision support | How do patients obtain and evaluate health information? | Internet use, trust in information sources, previous decision-support tools |
| Digital environment | Individual-level equity determinants: Digital capability and access | What difficulties do older adults face when using technology-based health tools? | Digital literacy, confidence, device access |
|  | Individual-level equity determinants: Differential benefit and exclusion | Could AI tools make participation more difficult for some patient groups? | Low literacy, low income, limited digital access |
|  | Interpersonal-level equity determinants : Trust and relationships | How might overreliance on AI affect patient-clinician trust? | Trust, communication, role of healthcare professionals |
|  | Interpersonal-level equity determinants: Team-based support | How can healthcare professionals collaborate to support patients’ use of AI tools? | Roles of nurses, physicians, social workers |
|  | Community-level equity determinants : Community support and infrastructure | Does the community have sufficient infrastructure to support older adults’ use of AI tools? | Wi-Fi, devices, technical assistance |
|  | Community-level equity determinants : Community partnerships | Which organizations could help promote use and education regarding AI tools? | NGOs, community centres, patient groups |
|  | Societal-level equity determinants : Policy and implementation | What policy or system-level factors may influence implementation of AI tools? | Regulation, governance, healthcare system support |

## Note: Interview guides were organized around two broad sections: (1) experiences of chronic disease management, information use, and shared decision-making in healthcare settings, and (2) perceptions of AI-PDAs and digital health equity considerations in digital environments. Determinants identified from the healthcare environment discussions were subsequently mapped to the individual, interpersonal, community, and societal levels of the DHEF during analysis. The healthcare environment section was intentionally designed to be more narrative and inductive, whereas the digital environment section incorporated more structured DHEF-informed probes because AI-PDAs represented a hypothetical intervention, and specific equity domains needed to be explored explicitly.
