# Supplementary material for: Operationalizing Digital Health Equity in Artificial Intelligence–Enabled Patient Decision Aids for Older Adults: Mixed Methods Study
Source: J Med Internet Res. 2026 Jun 29;28:e89011. doi: 10.2196/89011 (PMC13365886; doi:10.2196/89011)
Supplement: Multimedia Appendix 2 [file jmir_v28i1e89011_app2.docx]

**Supplementary Table S2.** Search algorithms of the umbrella review

|  | Paper | Suggestions related to digital health equity |
| --- | --- | --- |
| 1 | Trends and Gaps in Digital Precision Hypertension Management: Scoping Review | 1. [Individual level]: Consider **physical environment or digital literacy data** for personalized digital interventions, to promote health equity. 2. [Interpersonal level]: Employ hybrid digital health interventions designs combining human-driven consultation with automated elements. 3. [Cross-level-technology ] Incorporation of **diverse data sources,** such as race and ethnicity data, in personalized digital interventions for hypertension.  4. [Cross-level-technology ] ] Use reporting guidelines such as CONSORT-AI (Consolidated Standards of Reporting Trials–Artificial Intelligence) or TRIPOD-AI (Transparent Reporting of a Multivariable Prediction Model for Individual Prognosis or Diagnosis–Artificial Intelligence). |
| 2 | Examining Identity as a Variable of Health Technology Research for Older Adults: A Systematic Review | 1. [Cross-level-technology] Eliminate “othering" by **including identity dimensions of all older adult samples.** 2. [Cross-level-technology] Embrace the **full humanity of research participants**: Normalizing pro- viding more identity dimensions than just age and gender may help us to consider a more humanistic view of older adults engaged within HCI research.  3. [Cross-level-technology] Clearly **identifying the limitations of the study sample.** 4. [Cross-level-technology] Identifying **the impact of chosen methods on equity**. |
| 3 | AI Impact on Health Equity for Marginalized, Racial, and Ethnic Minorities | 1. [Cross-level-technology] **Algorithm Adjustment Techniques** during data pre-processing, in-processing, and post-processing. 2. [Cross-level-technology] **Improved Data Collection and Sampling** practices such as more detailed recording of race, ethnicity, gender identity, and other demographic factors in electronic health records to enable disaggregated analyses in AI systems; incorporating complementary information on social determinants through community partnerships to provide context for disparities. 3. [Societal level] Participatory techniques, such as forming inclusive design teams, **involve diverse stakeholders** and disciplines beyond computer sciences**.**  4. [Individual level] Promote participatory design processes that place **marginalized communities** at the core of AI system development and governance, ensuring equity and justice. |
| 4 | Digital Health Interventions for Hypertension Management in US Populations Experiencing Health Disparities A Systematic Review and Meta-Analysis | 1. [Community level] A multifaceted approach that combines diverse recruitment strategies with culturally tailored interventions—such as leveraging **faith-based community partnerships** and **incorporating motivational coaching grounded in individual belief systems**—offers a targeted means of reducing disparities and advancing digital health equity. 2. [Community level] Involve community health workers (CHWs) in interventions. 3. [Individual level] Whether the intervention was offered in **participants’ native or preferred language and included culturally sensitive messaging**, both of which are critical for improving accessibility to digital health interventions. |
| 5 | Machine learning in precision diabetes care and cardiovascular risk prediction | 1. [Cross-level-technology] Incorporate **real-time data and update model parameters**. 2. [Cross-level-technology] Use **explainable AI**, such as Local Interpretable Model-agnostic Explanations (LIME) and SHapley Additive exPlanations (SHAP). 3. [Cross-level-technology] Evaluating **model performance** across important subgroups, and incorporating input from key stakeholders and patient representatives.  3. [Society level] Navigating the **regulatory framework** of digital health technologies to ensure both legal compliance and the protection of patient safety. |
| 6 | Bridging the digital divide: artificial intelligence as a catalyst for health equity in primary care settings | 1. [Community level] **Community engagement** involves integrating community members into governance, including them in algorithm design and evaluation, and ensuring they have ownership or control over data generated from their communities. 2. [Societal level] The need for transparency and explainability calls for immediate **policy focus**. 3. [Cross-level-technology] Adopting AI technologies should be mandated to perform thorough **equity impact assessments** prior to deployment and maintain continuous monitoring throughout the implementation process. 4. [Cross-level-technology] **Professional training** programs should target the significant knowledge gaps that healthcare providers have concerning AI capabilities, limitations, and bias recognition.  5. [Cross-level-technology] Tackle various types of bias at once while ensuring that efforts to **reduce bias** do not unintentionally introduce new forms of discrimination. 6. [Cross-level-technology] Future AI development should emphasize the **full incorporation of social determinants of health data** to support more effective and holistic healthcare interventions. 7. [Societal level] **Government support** for AI in healthcare should emphasize **equitable development and prioritize tools** that serve disadvantaged populations. 8. [Societal level] Global cooperation and knowledge exchange should be encouraged to ensure that progress in equity-oriented AI contributes to worldwide health improvement. |
| 7 | Participatory AI Considerations for Advancing Racial Health | 1. [Individual level] An important aspect of participatory AI research is supporting marginalized communities in reflecting on how they want AI tools to influence their **emotional experiences** in healthcare, understanding both the potential benefits and risks, and identifying strategies to minimize any negative effects.  2. [Interpersonal level] Embrace **relationalism** as a driving concept in design eforts. 3. [Community level] Ensuring that **communities** are engaged in all phases of AI development. 4. [Cross-level-technology] **Trust** can be fostered through transparent information and clarity about its creators, while also recognizing the skepticism and past negative experiences that marginalized populations may have had with healthcare systems and AI. |
| 8 | Opportunities and Challenges for Large Language Models in Primary Health Care | 1. [Societal level] Additionally, it is essential to fully leverage **the role of county-level medical communities** to promote the descent of high-quality medical resources. 2. [Community level] Promote **community outreach programs** that encourage family-supported and technology-assisted self-health management to prevent disease and advance health equity. 3. [Community level] Engage communities in longitudinal design on their terms. 4. [Cross-level-technology] It is essential to incorporate **cross-cultural datasets** and develop intelligent models that accommodate different dialects and the needs of older adults. 5. [Societal level] It is recommended to include medical professionals (e.g., dentists, traditional Chinese medicine practitioners, rehabilitation doctors) and non-medical personnel (e.g., psychological counselors, community workers) for **interdisciplinary collaboration**. |
| 9 | Facilitators and barriers affecting the implementation of e-health for chronic respiratory diseases in remote settings: a qualitative evidence synthesis | 1. [Individual level] General comfort with technology and training requirements can **vary across groupsolder patients** and those with lower education levels often needed more time to learn.  2. [Community level] Adapting e-health interventions via symbolism, traditional medical practices to the local **communities**. 3. [Societal level] Financial considerations emerged as major barriers at multiple levels in most studies. Ensuring that interventions are financially **accessible** is essential for successful e-health implementation. |
| 10 | The Efficacy of Health Information Technology in Supporting Health Equity for Black and Hispanic Patients With Chronic Diseases: Systematic Review | 1. [Interpersonal level] **Caregivers and other members** of the patient’s network should be incorporated into the design of health information technology.  2. [Cross-level-technology] It is crucial to specifically **recruit and investigate older adults from racial minority groups**, as they are less likely than their non-minority counterparts to use health management websites or search the web for health information to support chronic disease self-management. 3. [Cross-level-technology] More research is needed to examine **whether and how access to and use of health information technologies influence** adherence to recommended self-management behaviors and subsequent outcomes. |
| 11 | 25 Years of Digital Health Toward Universal Health Coverage in Low- and Middle-Income Countries: Rapid Systematic Review | 1. [Societal level] **Joint initiatives** between governments and technology companies to develop scalable digital health solutions. 2. [Societal level] Implement targeted health care policies that reflect **sociocultural norms and practices**. 3. [Community level] Community-level measures such as expanding affordable internet access, strengthening local digital infrastructure, and implementing community-based digital literacy programs are essential to ensure equitable access to digital health tools, particularly for economically disadvantaged populations. 4. [Community level] Engaging and **empowering the community** (e.g., empowering local health workers to train communities) is critical to the successful implementation of digital health. |
| 12 | Artificial Intelligence in Chronic Disease Management for Aging Populations: A Systematic Review of Machine Learning and NLP Applications | 1. [Cross-level-technology] It is essential to train models using **diverse datasets**, across different populations, medical institutions, or data distributions.  2. [Interpersonal level] **Clinician adoption** is critical for the successful implementation of AI tools. 3. [Cross-level-technology] Developing and **training the model** with data that adhere to clinical guidelines, while ensuring continuous evaluation and adjustment during its application. 4. [Societal level] Organizations must also conduct regular compliance reviews, employee training, and **data protection** impact assessments.  5.[Societal level] Seamless **integration** with existing healthcare systems is essential for the effective implementation of AI tools. 6. [Individual level] AI-powered tools provide accessible and interactive **psychological support**, enhancing traditional therapy and enabling continuous care.  7. [Cross-level-technology] **Transparent and explainable AI systems**, and diverse, representative training data are crucial to ensure equitable and responsible use of AI. |
| 13 | Artificial intelligence in diabetes management: Advancements, opportunities, and challenges | 1. [Cross-level-technology] AI algorithms must be **trained on fair datasets** that include and accurately represent so- cial, environmental, and economic factors that influence health. |
